# The Effect of the Direction of Primary Lateral Spinal Curvature on Postural Stability in Children with Scoliosis

**DOI:** 10.3390/jcm13061690

**Published:** 2024-03-15

**Authors:** Andrzej Siwiec, Małgorzata Domagalska-Szopa, Ilona Kwiecień-Czerwieniec, Andrzej Szopa

**Affiliations:** 1 John Paul II Pediatric Center in Sosnowiec, 41-218 Sosnowiec, Poland; 2 Department of Developmental Age Physiotherapy, Medical University of Silesia in Katowice, Medyków 12, 40-752 Katowice, Poland; 3 Department of Physiotherapy, Medical University of Silesia in Katowice, Medyków 12, 40-752 Katowice, Poland; 4 Neuromed, Rehabilitation and Medical Center, Armii Krajowej 34, 40-698 Katowice, Poland

**Keywords:** scoliosis, body weight distribution, posturometric test

## Abstract

**Background:** The purpose of the present study was to determine the impact of the direction and magnitude of primary lateral spinal curvature in children with scoliosis. **Methods:** Ninety-six children diagnosed with scoliosis were included in the study group, and fifty healthy peers were included in the control group. Posturographic measurements of body weight distribution and posturometric tests with eyes open and closed were performed. **Results:** Based on the symmetry index values, the study group was divided into children with symmetrical and asymmetrical body weight distributions on the basis of support. Then, taking into account the direction of the primary curvature, children with asymmetrical body weight distributions were divided into: (1) children with left-sided or right-sided scoliosis with overload on the same side of the body; and (2) children with left-sided or right-sided scoliosis with overload on the opposite side of the body. According to both posturometric tests, increased CoP spatial displacement was observed in the children with scoliosis compared to the healthy controls. The obtained results showed that increased asymmetry index and Cobb angle values significantly increase medial–lateral postural instability in children with scoliosis. **Conclusions:** These findings suggest that treatment to restore symmetric body weight distribution may prevent the progression of postural instability; however, this requires confirmation through further investigation.

## 1. Introduction

Although scoliosis results in lateral curvature of the spine, it is actually a more complex, three-dimensional structural deformation of the spine, which, apart from deformation in the “coronal” (frontal) plane, also involves the sagittal and axial planes [1]. Most scoliosis cases are of unknown origin, hence the name idiopathic scoliosis (IS).

Although the causes of scoliosis are not yet known, there are many factors involved in its etiology [2,3].

Assuming that, irrespective of the primary triggers of IS, persistent asymmetric mechanical loading relative to the spinal axis may influence scoliosis development and progression, several recent studies have focused on analyzing body weight distribution (BWD) on the basis of support (BoS) between body sides [4,5,6,7,8] and assessing postural stability in children/adolescents with scoliosis [9,10,11,12,13,14,15,16,17,18,19,20,21,22,23,24].

A review of studies on postural stability in children/adolescents with scoliosis in comparison with their typically developing peers revealed that the results were not fully consistent. Notably, both earlier studies [8,9,10] as well as recent studies [14,15,16,17,18,19,20,21] reported significant differences in stability, while others reported a lack of differences in stability [22,23,24,25,26].

In addition, the cited literature focuses on the impact of various clinical features of scoliosis on postural stability. Gauchard et al. [10] and Wiernicka et al. [11] evaluated the effect of different types of progressive idiopathic scoliosis, and Park et al. [12] evaluated the effect of the scoliosis angle on static and dynamic postural control [9]. Allard et al. [13], Leteneur et al. [14], and Nault et al. [16] focused on the effect of body somatotype on standing balance. However, none of the studies conducted thus far have assessed the effect of the direction of primary lateral spinal curvature on standing stability in children with scoliosis.

Assuming that examining the distribution of body weight to address the direction of the primary curvature of the spine may have revealed some new dependencies in our previous pilot study, we examined BWD between body sides corresponding to convex and concave scoliotic curves [16]. These results seemed so promising that they encouraged us to further research postural static stability in children with IS, accounting for the direction of the primary curvature of the spine. Therefore, the first aim of the current research was to verify the hypothesis on the occurrence of postural instability in children with IS, and the second was to determine the impact of the direction and magnitude of primary lateral spinal curvature on standing stability in these children [15].

## 2. Materials and Methods

### 2.1. Ethics Statement

The present study is part of a larger research project called “Postural control in children with scoliosis” and involves the assessment of BWD in field studies.

The current research was performed in accordance with the Declaration of Helsinki and was approved by the Bioethical Committee of Silesian Medical University (PCN/0022/KB1/86/20/21). All parents/guardians gave their written informed consent prior to the subjects’ enrollment in the study.

This was a cross-sectional observational study.

### 2.2. Material

Ninety-six children diagnosed with scoliosis by an orthopedist based on X-ray images and who were cared for at a local orthopedic outpatient clinic, aged 7–11 years (9.5 ± 2.1), were included in the study. The main inclusion criteria were as follows: (1) children (male and female) aged 7–11 years (period of modest mid-growth spurt) who had undergone X-ray photography in the frontal view or agreed to be X-rayed for Cobb angle measurements before the start of the experiment; (2) children with IS treated conservatively without brace treatment (Cobb’s angle = 11°~25° in the major curve); and (3) children with the ability to understand and follow instructions.

The diagnosis and severity of IS were determined according to the Cobb’s angle, which was measured on X-ray photographs in the frontal view by a radiologist who was blinded to the research project.

Fifty sex- and age-matched healthy controls were included in the control group. The exclusion criteria for both groups included (1) other acute and chronic diseases in addition to scoliosis, (2) asymmetry of the absolute length of the lower limbs, (3) spine or lower-limb surgery, and (4) lack of informed consent from the child’s parents or legal guardians to participate in the study.

The study group consisted of the same subjects who participated in our previous research project on the weight-bearing distribution between body sides corresponding to convex and concave scoliotic curves [15]. The characteristics of the study and control groups are presented in Table 1. There was no difference between the groups in terms of anthropometric features (Table 1).

### 2.3. Method

The examination consisted of two parts: (1) posturographic BWD distribution and (2) posturographic testing with open and closed eyes. First, the BWD distribution was measured, and then, two posturographic tests of center of pressure (COP) measurements were taken, i.e., in the eyes-open and eyes-closed conditions. During both tests, the participants stood barefoot on a force platform (situated on the ground) in a natural position with their arms hanging loosely at their sides. Each of these measurements took 30 s. Each measurement included 3 trials lasting for 30 s with a 30-second pause between trials. The average values of the results of these three trials were analyzed. A force platform (PDM Multifunction Force Measuring Plate, Zebris, Isny, Germany) was used for all of the measurements.

The following posturometric parameters were analyzed: (1) BWD between the right and left sides of the body; (2) COP displacement: mean of points with x-coordinates (medial–lateral direction of the COP), mean of points with y-coordinates (posterior–anterior direction of the COP), sway path length of the COP; and (3) the COP area (the area of the ellipse containing 95% of the recorded points of the projection of the COP into the ground, the length of the short axis of the ellipse (the width of the ellipse), and the length of the long axis of the ellipse (the height of the ellipse)).

### 2.4. Statistical Analysis

The symmetry index among children from the control group was obtained from the formula SI = QR − QL, where QR was the percentage load on the right side of the body, and QL was the percentage load on the left side. For statistical analysis purposes, the results of the percentage BWD on the BoS in children with scoliosis are presented in relation to the so-called convex and concave sides, replacing the division into the left and right sides of the body.

On the basis of the mean values and standard deviation of BWD among children from the control group, the symmetry index was calculated. The SI value based on the calculation of the Z-standardized results was taken as the cutoff point for the apparent asymmetry. In addition, 11.5% was used as the cutoff point and all values above this threshold were included. The boundary values of symmetry were determined as 50% ± 11%. Based on the symmetry index value, subjects in the study group were divided into children with symmetrical BWD on the BoS and children with asymmetrical BWD. Then, taking into account the direction of the primary curvature, the children with asymmetrical BWD on the BoS were divided into the following subgroups: children with left-sided scoliosis with overload of the right side of the body and children with right-sided scoliosis with overload of the left side of the body, who were classified as children with overload of the concave side of the body (CC); and children with left-sided scoliosis with overload of the left side of the body and children with right-sided scoliosis with overload of the right side of the body, who were classified as children with overload of the convex side of the body (CV).

Statistical analysis was carried out using the IBM SPSS Statistics 27 program. This was used to analyze basic descriptive statistics along with the Shapiro–Wilk test. A regression model was used to assess the impact of SI and Cobb angle on posturometric parameters. To compare the results of the posturographic tests conducted during a free-standing position with open and closed eyes between the control group and the study group, the Mann–Whitney U test was used; for comparisons among the study subgroups (S; CC; CV), the Kruskal–Wallis test was used. Multiple comparisons were performed using the Tukey’s *p*-value.

In the planning process of our study, the sample size was set to 96 participants, a decision primarily driven by the time constraints of the project. To ensure the methodological robustness of our approach, we conducted a detailed sensitivity analysis. The aim of this analysis was to determine the size of the potential effect that could be observed with the assumed sample size. For the above analyses, a sensitivity analysis was performed in the G*Power 3.1.9.4 program, assuming 95% of the statistical test power, α = 0.05 and *N* = 96. This analysis indicated an effect size f = 0.40, η^2^ = 0.06. Additionally, to assess our study’s ability to detect significant effects, we conducted a post hoc power analysis using the G*Power software. The results of this analysis indicated that for a regression analysis with an assumed *N* = 96 and H1 slope = 0.009, the power reaches 99%.

## 3. Results

Subjects in the study group were divided according to their symmetry index value and the direction of their primary curvature into the following subgroups:

(1) children with symmetrical BWD on the BoS (S; *n* = 20; 21%);
(2) children with asymmetric BWD on the BoS overloading the convex side (CV; *n* = 49; 51%);
(3) children with asymmetric BWD on the BoS overloading the concave side (CC; *n* = 27; 28%).

Interestingly, as many as 78% of the children with asymmetric BWD on the BoS overloading the concave side were children with long thoracic scoliosis.

Excluding the obvious significant differences in the asymmetry indices between the subgroup of children with symmetrical and asymmetrical BWD on the BoS, statistically significant differences were also found between them in terms of the primary curvature angle (Table 2). The value of the primary curvature angle (Cobb angle) on average was lowest in children with symmetrical BWD (S) and the angular value of curvature in children with overload on the convex side of the body (CV) was statistically greater than children with overload on the concave side (CC) (Table 2). No significant differences were observed in terms of the asymmetry index between the children with overloads on the convex and concave sides of the body (Table 2).

The comparative analysis showed no differences in terms of sex distribution between the subgroups (χ^2^(2) = 0.12, *p* = 0.944). There were also no significant differences in height distribution between the subgroups (F(2, 95) = 2.77, *p* = 0.68) or in age (F(2, 95) = 2.28, *p* = 0.108).

A comparison of the posturographic parameters between the control group and the study group showed that the children with scoliosis presented greater spatial COP parameters (the COP range of both medial–lateral (width of the ellipse) and anterior–posterior (height of the ellipse) displacement of the COP), and thereby greater sway area of the COP, in both conditions with open and closed eyes (Table 3).

Regression analyses were performed to examine the relationship between the parameters of both the posturometric tests (with eyes open and closed) and two potential predictors, i.e., the asymmetry index value and the primary curvature angle value (Cobb angle). Table 4 summarizes the descriptive statistics and analysis results for posturometric parameters in the eyes-open measurement, while Table 5 shows these for the eyes-closed measurement. As can be seen, the width of the ellipse of the COP area and the standard deviation of the y-axis of COP displacement obtained in both tests (with open and closed eyes) positively and significantly correlated with the criterion while controlling for age and sex. Both the symmetry index and the Cobb angle values significantly explain the variability of the width of the ellipse of the COP area and standard deviation of the y-axis of COP displacement obtained in both tests with open and closed eyes (for 26% and 16%, respectively). More details on the regression models applied may be found in Table 4 and Table 5, below.

The division of the patients with scoliosis into subgroups according to their symmetry index values and the direction of their primary curvature allowed us to obtain interesting results. A statistical comparative analysis between the subgroups of children with scoliosis revealed intergroup differences for three posturometric parameters, i.e., the path length of the COP, width of the ellipse of the COP area, and standard deviation of the y-axis of COP displacement, obtained in both tests with open and closed eyes (Table 6). Post hoc analysis (Tukey method) showed that all of these parameters were significantly lower in children with symmetrical (S) vs. asymmetrical BWD on the BoS (CV; CC) (Table 6).

Moreover, post hoc analysis revealed that the path length of the COP in children with asymmetrical BWD who had an overload of the concave side of the body (CC) was greater than that in those who had an overload of the convex side (CV) (Table 6).

## 4. Discussion

The objective of this project was to answer two questions: (1) are there postural stability deficits in children with scoliosis compared to their healthy peers, and (2) are the parameters of standing stability in these children related to the direction and magnitude of their primary lateral spinal curvature?

For this purpose, we partially used the results of our previous study, which examined BWD between the body sides in children with scoliosis [15]. This study confirmed that 80% of the study group of children with IS presented with an asymmetrical BWD on the BoS in comparison with their heathy peers [15]. Although these results may seem not very original, they are based on thorough statistical analyses, which allowed us to define the boundary values of the broad norm of symmetry of the BWD between the sides of the body in healthy peers [15]. Here, we defined the boundary values of symmetry, which can be commonly used in studies on BWD between body sides as the criterion of symmetry of the BWD between body sides. Using the percentage BWD on the BoS in relation to the convex and concave sides of the curvature replaced the commonly used approach of dividing into the left and right sides of the body. This approach to the analyzed problem used in the present study allowed us to more accurately recognize the number of children with scoliosis with an asymmetrical BWD (80% of the target population). Moreover, this method allowed us to distinguish children with asymmetric body weight overloading the convex side of their body from children with overload on the concave side. Because our research included children with scoliosis, these results fully confirmed previous reports on the effect of asymmetrical BWD on the BoS in children with IS when standing [4,5,6,7,14,17]. Although it would have been very interesting at this stage of our project research, it was not possible to identify any demographics or clinical characteristics that would determine the direction of overloading on the convex or concave sides of the body.

Although previous investigations on postural control in children/adolescents with scoliosis compared to healthy controls have reported inconsistent findings [24], our results confirmed impaired postural stability in children with scoliosis. In both sets of posturometric test results, i.e., with open and closed eyes, increased COP displacement was observed in the scoliosis group compared to the healthy control group. According to both sets of posturometric measurements, the scoliosis group exhibited greater spatial COP parameters (COP range of both medial–lateral and anterior–posterior displacement of the COP), and thereby greater sway area of the COP; however, there was no significant difference between the children with scoliosis and the controls in terms of the COP path length or COP velocity. These results are fully consistent with the findings of Gruber et al. [18]; however, numerous studies have reported not only a greater COP range in the medial–lateral and anterior–posterior directions and sway area but also a greater sway path length in children/adolescents with scoliosis [5,10,12,19,20]. The enlarged values of spatial COP displacement parameters in both directions (medial–lateral and anterior–posterior occurring at normal excursions and COP velocity, as found in the present study) may result in large body oscillations in all directions and indicate decreased spatial postural stability in scoliosis patients [21,23]. Although there is no consensus in the literature on postural instability in scoliosis patients, our results are compatible with the results of most recent studies [12,13,14,15,16,17,18,19,20,21] as well as with the findings reported in both the latest systematic review and meta-analysis [23,27], which concluded that children and adolescents with IS were characterized by worse postural stability than healthy children were.

Although Wiernicka et al. [11], Dąbrowka et al. [25], and De Santiago et al. [26] showed that the sway path and area of the COP in a static standing test in the eyes-closed condition were greater than those in the eyes-open condition in scoliosis patients compared with healthy peers, in our study, there were no increases in the deprivation of visual inputs observed in either the group of IS adolescents or healthy adolescents. As in several other studies, the values of postural control variables in our study did not increase in the eyes-closed condition in either group, which reflects the role of vision in the participants’ postural control [10,11,12].

However, the main purpose of the present study was to determine the impact of the magnitude and direction of primary lateral spinal curvature on standing stability in these children. The results of the regression analysis confirmed that increased symmetry index and Cobb angle values caused significantly increased medial–lateral postural instability in children with IS, which manifested in a larger width of the ellipse and standard deviation of the y-axis.

The division of the subjects with scoliosis into subgroups according to the symmetry index and the direction of their primary curvature allowed us to solve the problem of recognition the impact of the direction of primary lateral spinal curvature on standing stability in children with IS. Assuming that various types of asymmetric loads on the spinal axis (on the convex and concave body sides) in scoliosis may affect postural stability in different ways, the postural stability parameters were compared among three subgroups of children with scoliosis: children with symmetrical BWD on the BoS (S), and children with asymmetric BWD overloading the CV and CC body sides. Comparative analysis between these subgroups revealed differences in the path length of the COP trajectory and medial–lateral displacement, which were observed in the width of the ellipse and standard deviation of the *y*-axis obtained in both the eyes-open test and eyes-closed test.

These results showed that both postural stability parameters, the COP trajectory (SPL) and medial–lateral displacement (width of the ellipse and standard deviation of the *y*-axis), were significantly lower in children with symmetrical BWD on the BoS (S) vs. both groups of children with asymmetrical BWD on the BoS (CV; CC). Considering that lower values of these parameters are thought to indicate enhanced postural stability, children with symmetrical BWD present the best conditions for postural stability. Interestingly, these parameters were significantly worse in children with asymmetrical BWD overloading the CC side than those in both the other subgroups. The enlarged values of the COP path length, COP velocity, and spatial COP displacement parameters in the mediolateral direction, as found in the present study in children with scoliosis with asymmetrical BWD overloading the CC side, may indicate that these patients have less spatial postural stability. Two potential reasons can be given to explain these observations. The first is the highest (on average) primary curvature angle (Cobb angle) value, and the second is the greatest value (on average) of asymmetry of BWD occurring in children with overload on the convex side of the body (CV) from all three subgroups of children with scoliosis. However, it is obvious that the impact of scoliotic spinal deformation on postural stability, according to the concept of the cone of economy, is multifactorial, and thus cannot be related only to radiographic static parameters such as the Cobb angle [28].

Due to the lack of similar studies in the relevant literature, comparison of our results with the results of other studies was not possible. This novel approach to the analysis of standing postural stability in children with scoliosis provides the first evidence that the presence of concave–convex biases may be a risk factor that influences postural stability deficits. Although the highest primary curvature angle (Cobb angle) and the greatest value of asymmetry of BWD on the BoS being in children with scoliosis overloading the convex side of the body may suggest the need to pay special attention to monitoring the progression of scoliosis in these children, the observation method of the present study does not allow us to draw such far-reaching conclusions.

We recognize that our study has some limitations. In the present study, parameters of postural control were investigated in relation to the direction and magnitude of primary lateral spinal curvature, i.e., in relation to the convex and concave sides of the curvature on standing postural stability. Unfortunately, the following factors were not included in this study of children with alterations in their spine curvature: the severity of the deformity and the magnitude of trunk rotation.

## 5. Conclusions

The results obtained in the present study showed that an increase in both asymmetry index and Cobb angle values in children with IS leads to significantly increased medial–lateral postural instability. On this basis, the following conclusion can be drawn: treatment aimed at restoring symmetric BWD in children with idiopathic scoliosis may prevent the progression of postural instability and may have an impact on the progression of scoliosis; however, this requires confirmation in a follow-up examination.

## Figures and Tables

**Table 1:** Characteristics of the control and study groups.

Parameters	Control Group (*n* = 50)	Study Group (*n* = 96)	*p*
	M (SD)	M (SD)	
Age (years)	9.5 (2.1)	9.3 (2)	0.741
Height (cm)	133 (12)	131 (12)	0.308
Body weight (kg)	33.2 (10.8)	32.4 (9.23)	0.777
BMI (kg/m^2^)	17.9 (3.1)	18.1 (2.7)	0.524
Sex, *n* (%)			
Girls	56 (58)	27 (54)	
Boys	40 (42)	23 (46)	

Abbreviations: M: mean value; *p*: statistical significance (Mann–Whitney U test); SD: standard deviation.

**Table 2:** The symmetry index (SI) and the value of the Cobb angle in individual subgroups of the overall study group. S—children with symmetrical BWD on the BoS (*n* = 20); CV—children with asymmetric BWD on the BoS overloading the convex side (*n* = 49); and CC—children with asymmetric BWD on the BoS overloading the concave side (CC; *n* = 27).

Parameters	S		CC		CV		K-W Test
	M (SD)	Range	M (SD)	Range	M (SD)	Range	
Symmetry index (%)	6.2 (3.1)	2–10	22.3 (9.1)	12–48	22.9 (10.0)	10–46	χ^2^(2) = 47.27, *p* < 0.001 ^ab^
Cobb angle (^o^)	15.5 (5.3)	10–25	17.8 (6.4)	10–25	22.9 (6.7)	12–25	χ^2^(2) = 16.81, *p* < 0.001 ^abc^
Age (years)	9.25 (2.1)	7–11	9.18 (2.1)	7–11	10.18 (1.9)	7–11	χ^2^(2) = 2.28, *p* = 0.108

Abbreviations: M: mean value; SD: standard deviation; K-W: Kruskal–Wallis test. Notes: ^a^ difference between S and CV; ^b^ difference between S and CC; ^c^ difference between CV and CC.

**Table 3:** Comparison of the results of posturographic tests conducted during independent standing in a free-standing position between the control group (*n* = 50) and the study group (*n* = 96).

Parameters	Eyes Open		Eyes Closed		p_1_	p_2_
	Control Group	Study Group	Control Group	Study Group		
	M (SD)	M (SD)	M (SD)	M (SD)		
MCoCx	17.76 (2.17)	17.70 (2.05)	17.82 (2.11)	17.98 (2.22)	0.773	0.707
MCoCy	22.71 (1.26)	22.80 (1.84)	22.77 (1.28)	22.84 (1.85)	0.418	0.425
SPL (cm)	112.74 (27.08)	125.62 (32.05)	112.80 (27.10)	125.66 (32.05)	0.123	0.126
SDx	0.61 (0.19)	0.63 (0.26)	0.67 (0.29)	0.67 (0.26)	0.905	0.866
SDy	0.47 (0.20)	0.54 (0.24)	0.53 (0.29)	0.58 (0.24)	0.097	0.174
WoE (cm)	3.23 (1.43)	4.12 (2.14)	**3.29 (1.46)**	**4.16 (2.14)**	**0.005**	**0.002**
HoE (cm)	4.39 (1.41)	5.20 (1.62)	**4.45 (1.49)**	**5.23 (1.62)**	**0.006**	**0.009**
AoE (cm^2^)	11.56 (6.31)	14.30 (8.15)	**11.62 (1.49)**	**14.34 (8.15)**	**0.004**	**0.004**

Abbreviations: MCoCx: mean of points with x-coordinates (medial–lateral direction) of the CoP; MCoCy: mean of points with y-coordinates (posterior–anterior direction) of the CoP; SPL: path length of the CoP; SDx: standard deviation of x; SDy: standard deviation of y; WoE: width of the ellipse; HoE: height of the ellipse; AoE: area of the ellipse. Notes: p_1_ value: statistical significance test result in the eyes-open condition; p_2_ value: statistical significance test result in the eyes-closed condition. Statistically significant differences are printed in bold (Mann–Whitney U test).

**Table 4:** Summary of regression models for posturometric parameters in the eyes-open measurement.

Dependent Variable	Predictors	B	SE	β	t	*p*	95% CI		R^2^
							LL	UL	
MCoCx	Intercept	18.72	1.15		16.25	<0.001	16.43	21.01	0.06
	SI (%)	0.02	0.02	0.08	0.71	0.476	−0.03	0.06	
	Cobb angle (^o^)	−0.09	0.04	−0.27	−2.33	0.022	−0.17	−0.01	
	Age	0.03	0.10	0.03	0.31	0.759	−0.17	0.24	
	Sex	0.11	0.43	0.03	0.25	0.801	−0.74	0.96	
MCoCy	Intercept	24.06	1.04		23.09	<0.001	21.99	26.13	0.01
	SI (%)	0.01	0.02	0.04	0.34	0.732	−0.03	0.05	
	Cobb angle (^o^)	0.01	0.04	0.02	−0.14	0.892	−0.07	0.06	
	Age	−0.17	0.09	−0.19	−1.82	0.073	−0.36	0.02	
	Sex	0.51	0.39	0.14	1.33	0.186	−0.25	1.28	
SPL (cm)	Intercept	89.96	17.75		5.07	<0.001	54.70	125.22	0.04
	SI (%)	0.28	0.34	0.10	0.84	0.403	−0.39	0.95	
	Cobb angle (^o^)	1.01	0.59	0.20	1.71	0.090	−0.16	2.16	
	Age	0.83	1.59	0.05	0.52	0.602	−2.32	3.98	
	Sex	5.72	6.58	0.09	0.87	0.387	−7.35	18.79	
SDx	Intercept	0.87	0.08		6.65	<0.001	0.40	0.74	0.07
	SI (%)	0.01	0.01	0.24	2.15	0.034	−0.01	0.11	
	Cobb angle (^o^)	−0.01	0.01	−0.03	−0.27	0.788	−0.01	0.01	
	Age	−0.03	0.01	0.26	−2.60	0.011	−0.06	−0.01	
	Sex	−0.03	0.05	−0.05	−0.53	0.599	−0.13	0.08	
SDy	Intercept	0.17	0.13		1.35	0.180	0.11	0.40	0.16
	SI (%)	0.01	0.01	0.27	2.55	0.012	0.01	0.01	
	Cobb angle (^o^)	0.01	0.01	0.23	2.17	0.033	0.01	0.02	
	Age	0.01	0.01	0.09	0.94	0.350	−0.01	0.02	
	Sex	−0.03	0.05	−0.05	−0.55	0.584	−0.12	0.07	
WoE (cm)	Intercept	0.70	1.04		0.67	0.504	−1.37	2.77	0.26
	SI (%)	0.07	0.02	0.39	3.90	<0.001	0.04	0.12	
	Cobb angle (^o^)	0.07	0.03	0.22	2.22	0.029	0.01	0.15	
	Age	0.06	0.09	0.06	0.63	0.528	−0.13	0.24	
	Sex	−0.13	0.39	−0.03	−0.34	0.735	−0.90	0.64	
HoE (cm)	Intercept	6.78	0.90		7.52	<0.001	4.99	8.57	0.03
	SI (%)	0.03	0.02	0.19	1.69	0.095	−0.01	0.06	
	Cobb angle (^o^)	−0.02	0.03	−0.07	−0.62	0.536	−0.08	0.04	
	Age	−0.17	0.08	−0.22	−2.15	0.034	−0.33	−0.01	
	Sex	−0.25	0.33	−0.07	−0.74	0.464	−0.91	0.42	
AoE (cm^2^)	Intercept	1.75	4.29		2.74	0.007	3.23	20.28	0.13
	SI (%)	0.28	0.08	0.38	3.48	<0.001	0.12	0.45	
	Cobb angle (^o^)	0.10	0.14	0.07	0.69	0.490	−0.19	0.39	
	Age	−0.48	0.38	−0.12	−1.26	0.211	−1.25	0.28	
	Sex	−0.29	1.59	−0.18	−0.18	0.855	−3.45	2.87	

Abbreviations: MCoCx: mean of points with x-coordinates (medial–lateral direction) of the CoP; MCoCy: mean of points with y-coordinates (posterior–anterior direction) of the CoP; SPL: path length of the CoP; SDx: standard deviation of x; SDy: standard deviation of y; WoE: width of the ellipse; HoE: height of the ellipse; AoE: area of the ellipse. Notes: B—beta, SE—standard error, β—differentiated beta, t—*t*-test coefficient, *p*—statistical significance of the *t*-test, LL—lower confidence interval, UL—upper confidence interval for beta, R^2^—specific r^2^ value, SI—symmetry index. Statistically significant values are printed in bold.

**Table 5:** Summary of regression models for posturometric parameters in the eyes-closed measurement.

Dependent Variable	Predictors	B	SE	β	t	*p*	95% CI		R^2^
							LL	UL	
MCoCx	Intercept	19.11	1.25		15.33	<0.001	16.63	21.59	0.02
	SI (%)	0.02	0.02	0.09	0.75	0.453	−0.03	0.06	
	Cobb angle (^o^)	−0.10	0.04	−0.27	−2.39	0.019	−0.18	−0.02	
	Age	0.03	0.11	0.03	0.31	0.760	−0.19	0.26	
	Sex	0.12	0.46	0.03	0.25	0.803	−0.80	1.03	
MCoCy	Intercept	24.10	1.04		23.13	<0.001	22.03	26.17	0.04
	SI (%)	0.01	0.02	0.04	0.34	0.732	−0.03	0.05	
	Cobb angle (^o^)	−0.01	0.04	−0.02	−0.14	0.892	−0.07	0.06	
	Age	−0.17	0.09	−0.19	−1.82	0.073	−0.36	0.02	
	Sex	0.51	0.39	0.14	1.33	0.186	−0.25	1.28	
SPL (cm)	Intercept	90.00	17.75		5.07	<0.001	54.74	125.25	0.04
	SI (%)	0.28	0.34	0.10	0.84	0.403	−0.39	0.95	
	Cobb angle (^o^)	1.01	0.59	0.20	1.71	0.090	−0.16	2.19	
	Age	0.83	1.59	0.05	0.52	0.602	−2.32	3.98	
	Sex	5.72	6.58	0.09	0.87	0.387	−7.35	18.79	
SDx	Intercept	0.91	0.14		6.48	<0.001	0.63	1.19	0.07
	SI (%)	0.01	0.01	0.24	2.15	0.034	0.01	0.11	
	Cobb angle (^o^)	−0.01	0.01	−0.03	−0.27	0.788	−0.01	0.01	
	Age	−0.03	0.01	−0.26	−2.60	0.011	−0.06	−0.01	
	Sex	−0.03	0.05	−0.05	−0.53	0.599	−0.13	0.08	
SDy	Intercept	0.30	0.12		1.67	0.099	−0.04	0.46	0.16
	SI (%)	0.01	0.01	0.27	2.55	0.012	0.01	0.01	
	Cobb angle (^o^)	0.01	0.01	0.23	2.17	0.033	0.01	0.02	
	Age	0.01	0.01	0.09	0.94	0.350	−0.01	0.03	
	Sex	−0.03	0.05	−0.05	−0.55	0.584	−0.12	0.07	
WoE (cm)	Intercept	0.74	1.04		0.71	0.480	−1.33	2.81	0.26
	SI (%)	0.08	0.02	0.40	3.90	<0.001	0.04	0.12	
	Cobb angle (^o^)	0.07	0.03	0.22	2.20	0.029	0.01	0.15	
	Age	0.06	0.09	0.06	0.63	0.53	−0.13	0.25	
	Sex	−0.13	0.39	−0.03	−0.34	0.735	−0.90	0.64	
HoE (cm)	Intercept	6.82	0.54		7.57	<0.001	5.03	8.61	0.03
	SI (%)	0.03	0.02	0.19	1.69	0.095	−0.01	0.06	
	Cobb angle (^o^)	−0.02	0.03	−0.07	−0.62	0.536	−0.08	0.04	
	Age	−0.17	0.08	−0.22	−2.15	0.034	−0.33	−0.01	
	Sex	−0.25	0.33	−0.08	−0.74	0.464	−0.91	0.42	
AoE (cm^2^)	Intercept	11.79	4.29		2.75	0.007	3.27	20.32	0.14
	SI (%)	0.28	0.82	0.38	3.48	<.001	0.12	0.45	
	Cobb angle (^o^)	0.10	0.14	0.08	0.70	0.490	−0.18	0.38	
	Age	−0.48	0.38	−0.12	−1.26	0.211	−1.25	0.28	
	Sex	−0.29	1.59	−0.02	−0.18	0.855	−3.45	2.87	

Abbreviations: MCoCx: mean of points with x-coordinates (medial–lateral direction) of the CoP; MCoCy: mean of points with y-coordinates (posterior–anterior direction) of the CoP; SPL: path length of the CoP; SDx: standard deviation of x; SDy: standard deviation of y; WoE: width of the ellipse; HoE: height of the ellipse; AoE: area of the ellipse. Notes: B—beta, SE—standard error, β—differentiated beta, t—*t*-test coefficient, *p*—statistical significance of the *t*-test, LL—lower confidence interval, UL—upper confidence interval for beta, R^2^—specific r^2^ value, SI—symmetry index. Statistically significant values are printed in bold.

**Table 6:** Comparison of the results of posturographic tests conducted during independent standing in a free-standing position among groups S—children with symmetrical BWD on the BoS (*n* = 20); CV—children with asymmetric BWD on the BoS overloading the convex side (*n* = 49); and CC—children with asymmetric BWD on the BoS overloading the concave side (*n* = 27).

Parameters	Eyes Open					Eyes Closed				
	M	SD	H (2)	*p*	η²	M	SD	H (2)	*p*	η²
MCoCx			0.64	0.727	<0.01			0.64	0.727	<0.01
S	17.49	1.98				17.75	2.16			
CV	17.89	2.09				18.20	2.27			
CC	17.52	2.07				17.75	2.23			
MCoCy			4.22	0.122	0.05			4.22	0.122	0.05
S	22.86	0.94				22.90	0.94			
CV	23.03	2.26				23.07	2.26			
CC	22.34	1.45				22.38	1.45			
SPL (cm)			7.05	0.029 ^abc^	0.04			7.05	0.029 ^abc^	0.04
S	114.97	28.38				115.01	28.38			
CV	121.45	27.39				121.49	27.39			
CC	141.08	37.57				141.12	37.57			
SDx			0.79	0.674	<0.01			0.79	0.674	<0.01
S	0.60	0.28				0.64	0.28			
CV	0.65	0.27				0.69	0.27			
CC	0.62	0.22				0.66	0.22			
SDy			10.81	0.004 ^ab^	0.51			10.81	0.004 ^ab^	0.50
S	0.41	0.23				0.45	0.23			
CV	0.56	0.22				0.60	0.22			
CC	0.62	0.25				0.66	0.25			
WoE (cm)			11.18	0.004 ^ab^	0.45			11.18	0.004 ^ab^	0.45
S	2.96	1.66				3.00	1.66			
CV	4.18	1.99				4.22	1.99			
CC	4.85	2.40				4.90	2.40			
HoE (cm)			0.01	0.994	<0.01			0.01	0.994	<0.01
S	5.06	1.67				5.10	1.67			
CV	5.29	1.62				5.33	1.62			
CC	5.14	1.63				5.18	1.63			
AoE (cm^2^)			3.72	0.156	0.06			3.72	0.156	0.06
S	12.07	8.66				12.11	8.66			
CV	14.30	7.91				14.34	7.91			
CC	15.97	8.11				16.01	8.11			

Abbreviations: MCoCx: mean of points with x-coordinates (medial–lateral direction) of the CoP; MCoCy: mean of points with y-coordinates (posterior–anterior direction) of the CoP; SPL: path length of the CoP; SDx: standard deviation of x; SDy: standard deviation of y; WoE: width of the ellipse; HoE: height of the ellipse; AoE: area of the ellipse. Notes: ^a^ difference between S and CV; ^b^ difference between S and CC; ^c^ difference between CV and CC. Kruskal–Wallis test.

## Data Availability

The datasets analyzed during the current research are available from the corresponding author on reasonable request.
